# Exercise combined with trimetazidine improves anti-fatal stress capacity through enhancing autophagy and heat shock protein 70 of myocardium in mice

**DOI:** 10.7150/ijms.53899

**Published:** 2021-02-06

**Authors:** Lingjun Jiang, Xuanlin Shen, Yaoshan Dun, Murong Xie, Siqian Fu, Wenliang Zhang, Ling Qiu, Jeffrey W. Ripley-Gonzalez, Suixin Liu

**Affiliations:** 1 Division of Cardiac Rehabilitation, Department of Physical Medicine & Rehabilitation, Xiangya Hospital Central South University, Changsha, Hunan 410008, P.R China.; 2 Department of Rehabilitation, The Affiliated Changshu Hospital of Xuzhou Medical University, Changshu, Jiangsu 215500, P.R China.; 3 Division of Sport and Rehabilitation Medicine, University Hospital Ulm, Parkstr. 11, 89075, Ulm, Germany.

**Keywords:** anti-stress capacity, Trimetazidine, exercise, autophagy, heat shock protein 70

## Abstract

**Background:** Anti-stress capacity is important to resist the occurrence of adverse events. To observe the effects of exercise, trimetazidine alone or combined on the anti-stress capacity of mice, and further explore its potential mechanism.

**Methods:** Forty-four C57BL/6 male mice aged 8 weeks were randomly divided into four groups (n=11 for each group): control group (group C), exercise group (group E), trimetazidine group (group T), exercise combined with trimetazidine group (group TE). After the intervention, each group was randomly subdivided into the exhaustive exercise (EE, n=6) and the non-EE (n=5) subgroups. The mice in the EE-subgroup underwent EE. Mice were sacrificed 12 hours later after EE. The myocardial ultrastructure and autophagosomes were observed under an electron microscope. The expression of autophagy-related proteins: BNIP3, LC3-II, and P62 were analyzed and the heat shock protein 70 mRNA transcription and protein expression were also investigated.

**Results:** Exercise or trimetazidine increased the expression of BNIP3, LC3-II, and heat shock protein 70, decreased the expression of P62 pre- and post-EE while the combination has the synergistic effect.

**Conclusion:** Exercise and trimetazidine, alone or combined enhanced the anti-stress capacity of mice significantly. The underlying mechanism may be associated with the promotion of autography and the expression of heat shock protein 70.

## Introduction

An organism's reaction and adaptability to its external environment has direct implications on its survivability and recovery from stresses, both environmental and internal. Within patients, anti-fatal stress capacity reacts to acute negative stimuli as a prognostic predictor of adverse events, including but not limited to myocardial infarction, acute coronary syndrome, and sudden death 1-3. Therefore, in recent years multiple studies have been conducted to investigate the interaction between anti-fatal stress capacity and adverse health conditions, and the efficacy and the utility of exercise in improving health outcomes in patients with cardiovascular disease 4-6.

In our previous study we found that the time to exhaustion in mice during a fatal stress test had been significantly increased through exercise training, trimetazidine ingestion, and more so by a combination of both 7, effectively showing that these interventions had been instrumental for the improvement of anti-fatal stress capacity. The mechanisms behind exercise and trimetazidine showed favorable synergistic effects in skeletal muscle, enhancing autophagy, and improved mitochondrial function in mice under fatal stress. Another of our studies explored the capability of aerobic exercise training in improving exhaustive exercise-induced stress, finding that this increased exercise capacity in mice and ameliorated exhaustive exercise-induced stress damage in skeletal muscles through elevated autophagy 7, 8. Therefore, anti-stress capacity may be related to the ameliorating skeletal muscle injury and promotion of autophagy in skeletal muscle. However, it has not yet been investigated whether the same effect would be seen in the myocardium.

Heat shock proteins (HSPs) expressed at low levels under normal physiological conditions show a significant induction in response to various stressors in both *in vitro* and *in vivo* models 9, 10. This protein family belongs to molecular chaperones and supports cell survival under fatal conditions 11, 12. For instance, chemical and physical cell stress factors, such as heat and oxidative stresses, ischemia, and lack of energy induce the expression of HSPs in various cell structures 13. Furthermore, under pathological conditions, HSPs prevents apoptosis and promotes the repair of damaged proteins in cells 14, 15, suggesting that HSPs may protect cells from stressors. One of the most prominent HSPs is the 70 kDa HSP (HSP70). HSP70 is a highly conserved and essential protein against stress 16. Strength training and voluntary running upregulate the expression of HSP70 17, 18. Autophagy is a complex pathway responsible for degrading and recycling damaged organelles and nutrients under stress states such as hunger, ischemia, or hypoxia 19-21. The activation of autophagy is an effective approach to resist these stress states. Therefore, it is likely that the cardioprotective effect of exercise and trimetazidine is related to autophagy. As mentioned above, our previous study found that exercise and trimetazidine synergistically improved anti-fatal stress capacity 7, however, no study has shown whether trimetazidine alone or with exercise improves anti-stress capacity through HSP70 and autophagy.

Therefore, in this study, we aim to explore the mechanism behind exercise and trimetazidine alone or combined improvement on anti-fatal stress capacity through autophagy and HSP70 expression of the myocardium.

## Materials and methods

### Animals and study design

Forty-four C57BL/6 male mice aged 8 weeks, weighing 22.66 ± 2 g, were randomly assorted into four groups (11 mice in each group): control (group C), exercise (group E), trimetazidine (group T), trimetazidine combined with exercise (group TE). All mice were fed adaptively for one week before the experiment. Saline intragastric administration, medium intensity swimming training, trimetazidine (10 mg/kg) intragastric administration, and medium intensity swimming training combined with trimetazidine intragastric administration were given respectively. After the intervention, each group was randomly subdivided into the non-EE and the EE subgroups. The mice in the EE subgroups underwent EE (details below). 12 hours after EE, the mice were euthanized under anesthesia. The feeding environment was clean, free diet, and the feeding temperature was constant (20 ± 2 °C). All animal experiments were performed with the approval of the Hunan Provincial People's Hospital Animal Care and Use Committee (code: SYXK 2015-0013).

### Aerobic exercise training protocol

As explained previously 7, mice in group E and group TE carried out adaptive non-weight-bearing swimming training at moderate intensity in a Morris water maze pool (type number: XR-XM101-R, 60 cm high, 120 cm in diameter) maintained at 30 ± 2 °C and a water depth of 30 cm. The time of swimming training was initially 10 minutes, thereafter the swimming time increased by 10 minutes every subsequent day until reaching 60 minutes per day. The training was maintained six days a week for 5 weeks.

### Exhaustive exercise

Mice in the EE subgroup were exposed to the fatal stress model 7. Before the exhaustive exercise, the body weight of each mouse was measured, and a load (5% of the weight) was tied to the tail and the mice swam to exhaustion. Exhaustion was classified as the point when the head of the mouse (especially the mouth and eyes) was immersed completely in the water lasting for 7 seconds at which point the experiment was terminated.

### Tissue processing

Anesthesia with 5% chloral hydrate (0.1 mL/10 g weight) was applied via injection and the mice were then euthanized after a blood sample was taken. The hearts of the mice were divided into two parts: one part (n=2 per group) was for the electron microscopy, and the other was placed in liquid nitrogen immediately, and then stored in a -80 °C refrigerator.

### Microstructure of myocardium under electron microscopy

To further scrutinize the anti-stress capacity and autophagy at the organelle level, the structure of the myocardium and mitochondria and autophagosome number in the myocardium (n=2 per group) were observed under electron microscopy (Tecnai G2 Spirit, FEI, USA).

### Western blot

Proteins were extracted from the heart, the following antibodies were used: BNIP3 (ab109362, rabbit, 1:1000) from Abcam (England); LC3-II and LC3-I (14600-1-AP, rabbit, 1:500), and P62 (18420-1-AP, rabbit, 1:1000) from Proteintech (USA); HSP70 (21206-1-AP, rabbit,1:500) from Proteintech (USA) and GAPDH (AP0063, rabbit, 1:5000) from Bioworld (USA); Protein expression was normalized to that of GAPDH.

### Quantitative real-time PCR

Total RNA was isolated with Trizol reagent (Invitrogen, United States), and reverse transcription of RNA was performed. First-strand cDNA was prepared with the cDNA Reverse Transcription Kit (ComWin Biotech, China). The thermal cycling was carried out with a program of 95 °C for 10 minutes, followed by 40 cycles with denaturation at 95 °C for 15 seconds, annealing, and elongation at 60 °C for 60 seconds. All samples were normalized relative to GAPDH levels. The primer sequences used in this study are shown in Table 1.

### Statistics

All experimental data were performed with GraphPad Prism 8.0.1 and the data was demonstrated as the mean and standard deviation (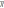
± SD). Using one-way ANOVA analysis of variance followed by a homogeneity of variance test to determine statistical significance.* P <0.05* was considered to indicate significance.

## Results

### Exercise and trimetazidine alone or combined, improved autophagy, and the expression of HSP70 in myocardium pre-EE

**Figure 1:**
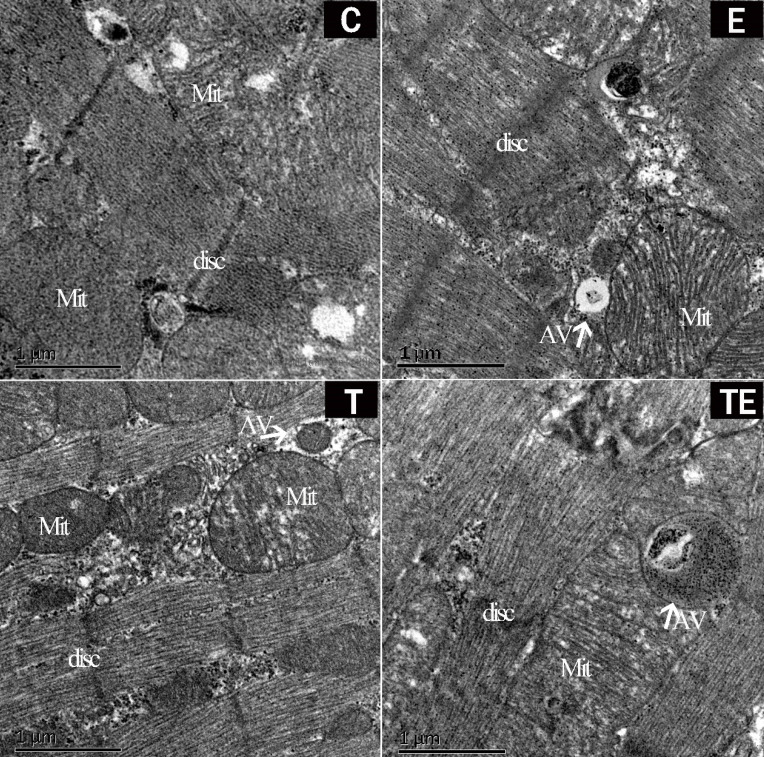
Representative changes in rat myocardium under electron microscope pre-EE. Exercise and trimetazidine alone or combined, improved mitochondrial autophagy pre-EE. The electron microscope shows that the mitochondria number myocardium was significantly increased in the E, T, and TE groups. Mit: mitochondria; AV: autophagic vacuole; disc: myocardial disc.

After 5 weeks of intervention, there were no autophagosomes in group C while autophagosomes were found in group E, T, and TE under electron microscopy (Fig. 1). The mitochondrial structure was clear and the myocardium was not damaged in each group.

**Figure 2:**
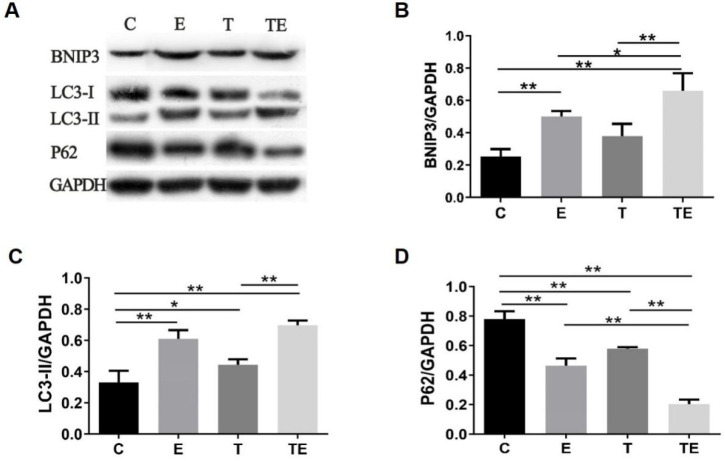
Exercise and trimetazidine alone or combined, improved mitochondrial autophagic protein expression pre-EE. The protein expression of BNIP3, LC3-II, P62 were detected respectively. Data is demonstrated as the mean ± SD (n=3). * P<0.05, **P<0.01.

**Figure 3:**
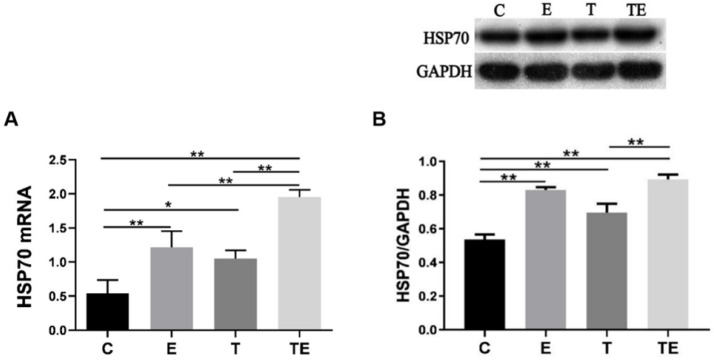
HSP70 response to exercise, trimetazidine, and exercise plus trimetazidine pre- EE. Exercise and trimetazidine alone or combined upregulated the expression of HSP70 pre-EE. Data is demonstrated as a mean ± SD (n=3). * P<0.05, **P<0.01.

The autophagy-related proteins are presented in Figure 2. Compared with group C, the expression of BNIP3 had increased in group E and TE significantly (*P*<0.01), LC3-II increased while P62 decreased in groups E, T, and TE. Compared with group E and T respectively, the expression of BNIP3 was upregulated while the expression of P62 was downregulated in group TE (*P*<0.05). Compared with group E, the expression of LC3-II in group TE was upregulated but there is no statistical significance (*P*=0.09). Compared with group C, HSP70 mRNA expressed higher in group E, T, and TE (*P*<0.01,* P*<0.05,* P*<0.01 respectively) (Fig. 3A). HSP70 mRNA in group TE was also higher compared with group E and T (*P*<0.01,* P*<0.01 respectively) (Fig. 3A).

### EE-induced myocardium damage was ameliorated by exercise combined with trimetazidine

**Figure 4:**
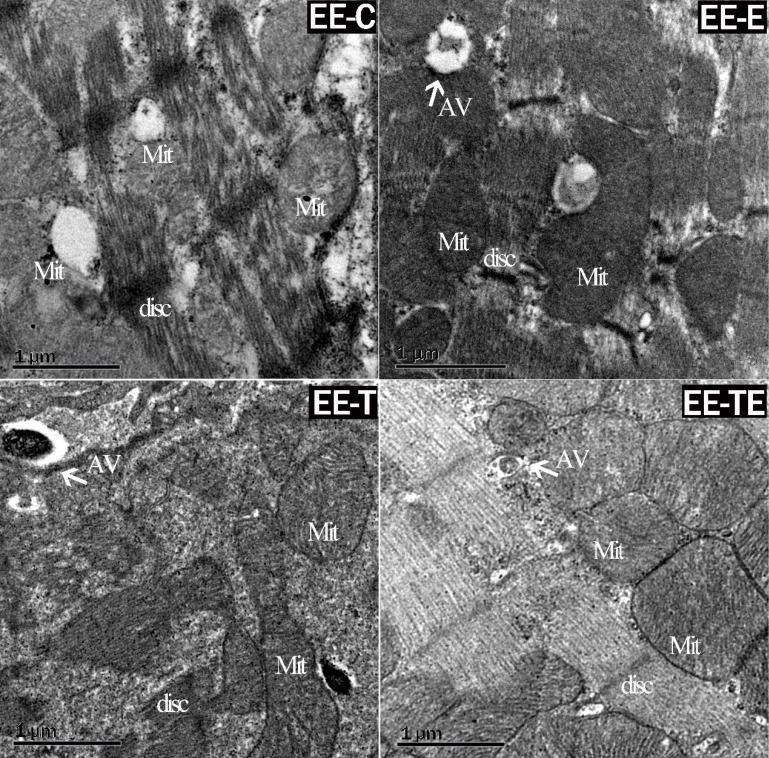
Representative changes in rat myocardium under electron microscope post- EE. Exercise and trimetazidine alone or combined, improved mitochondrial autophagy post- EE. Electron microscope showed that the mitochondria number was significantly increased in the E, T, and TE groups post- EE. Mit: mitochondria; AV: autophagic vacuole; disc: myocardial disc.

Upon inspection, electron microscopy showed cardiac muscle fibers were fractured, and mitochondria were edematous in group EE-C after EE. Autophagosome was found in group EE-E and EE-T. In group EE-TE, the myocardium was integrated, the mitochondrial structure was clear and autophagosome was found (Fig. 4).

**Figure 5:**
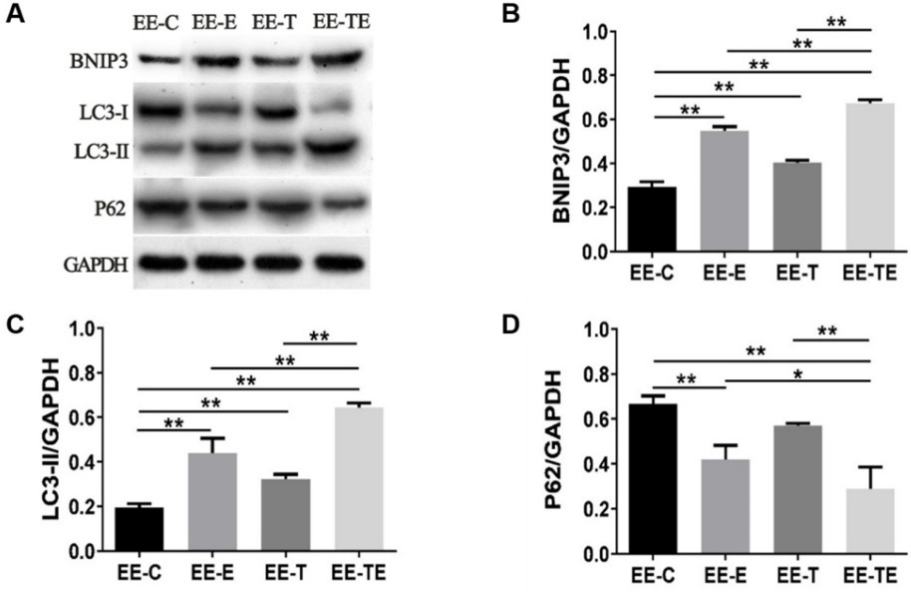
Exercise and trimetazidine alone or combined, improved mitochondrial autophagic protein expression at 12 hours post- EE. The protein expression of BNIP3, LC3-II, P62 were detected respectively. Data is demonstrated as the mean ± SD (n=3). * P<0.05, **P<0.01.

Compared with group EE-C, the expression of BNIP3, LC3-II all increased in group EE-E, EE-T, and EE-TE (*P*<0.01), as shown in Fig. 5B and Fig. 5C. The expression of P62 was decreased in group EE-E and EE-TE (*P*<0.01), though P62 was reduced in group EE-T, there was no significant difference (*P*=0.10) (Fig. 5D). Mice in the EE-TE group presented significantly higher expressions of BNIP3, LC3-II (*P*<0.01), and lower P62 than mice in group EE-E and EE-T.

**Figure 6:**
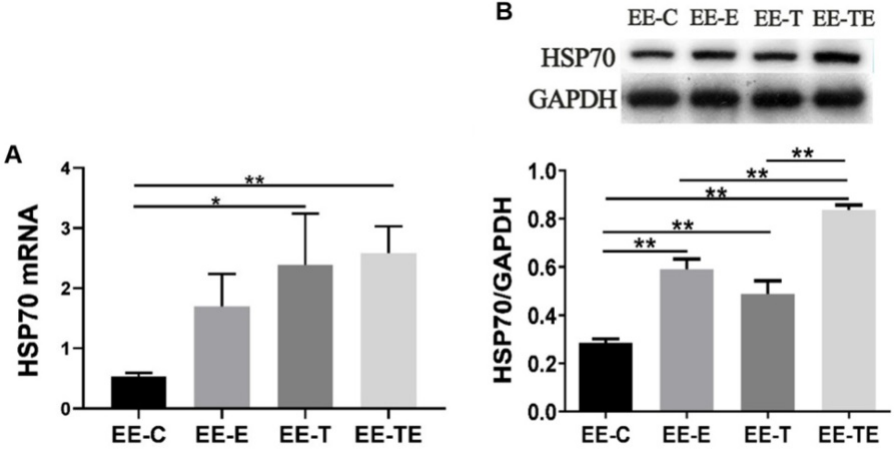
HSP70 response to exercise, trimetazidine, and exercise plus trimetazidine at 12 hours post- EE. Exercise and trimetazidine alone or combined upregulated the expression of HSP70. Data is demonstrated as the mean ± SD (n=3). *P<0.05, **P<0.01.

**Figure 7:**
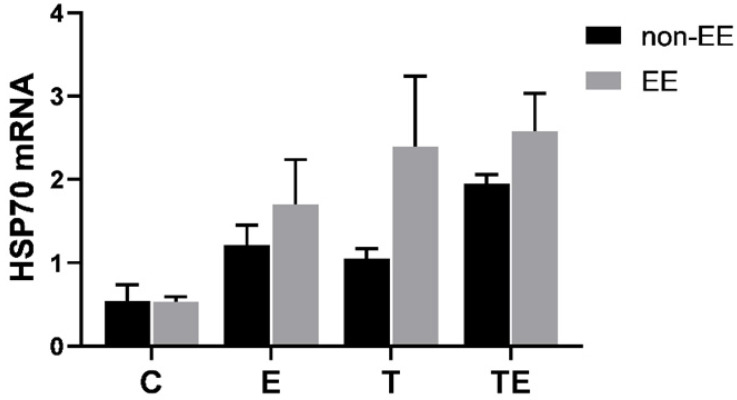
The comparison between the expression of HSP70 mRNA pre-and post- EE. Data is demonstrated as the mean ± SD (n=3). *P<0.05, **P<0.01.

HSP70 mRNA was upregulated in group EE-E, EE-T, and EE-TE compared with group EE-C (*P*=0.12,* P*<0.05,* P*<0.01 respectively). Compared with EE-E, EE-T respectively, HSP70 mRNA expressed higher in group EE-TE, but there were no statistically significant differences (*P*=0.28,* P*=0.97, respectively) (Fig. 6A). The HSP70 protein expression approached a trend for difference between pre-EE and post-EE but did not reach statistical significance (Fig. 7).

## Discussion

In our previous study, exercise and trimetazidine alone or combined, prolonged swimming time to exhaustion in mice (an average increase of 237% in those with trimetazidine, 275% in those who did exercise, and 520% in those with trimetazidine combined with exercise) and reduced the serum creatine kinase (CK) activity and skeletal muscle injury induced by the EE 7. In the current study, we observed that exercise and trimetazidine alone effectively improved the anti-stress capacity of mice and reduced the myocardial tissue injury caused by EE, and found that the combination of exercise with trimetazidine had an additive effect on enhancing anti-stress capacity. Through our research, it was found that the mechanism behind these improvements may be related to the up-regulation of autophagy and HSP70 of the myocardium.

Autophagy provides crucial protection against stress injury. EE as a fatal stress poses several kinds of damages: tissue injuries, inflammation, overproduction of reactive oxidative species (ROS), and mitochondrial dysfunction 22. EE increases ROS production in muscle, our previous studies have shown that exercise and trimetazidine/Rhodiola sacra alone or combined improved autophagy activity in skeletal muscle post-EE 7, 8. Enhanced autophagic responses allow the organism or cells to cope with the adverse stress 23-25. In consistence with previous studies 26-28, the findings here, showed that exercise and trimetazidine also promoted autophagy. Furthermore, trimetazidine pretreatment may also have a protective effect against hypoxia/reoxygenation (H/R) injury via the promotion of autophagy 29, with previous studies showing that the administration of trimetazidine enhanced autophagy to inhibit myocardial fibrosis and improved diabetic cardiomyopathy in mice 30. Our research found, for the first time, that the combination of exercise and trimetazidine has synergistic effects on upregulating autophagy in the myocardium of mice. We also found that the changes in autophagy are not only short-term responses to physical activity but also an adaptation to prepare the organism for the next activity, since 12 hours post-EE, exercise plus trimetazidine still enhanced the autophagy compared to exercise or trimetazidine alone. Activating autophagy improved the degradation and recycling of damaged proteins and organelles and ensured the normal function of myocardial cells. Thus, reduced myocardial ischemic hypoxia injury and enhanced myocardial degradation and recovery processes may limit the inhibition of autophagy levels in subsequent EE. That may be the reason why trimetazidine combined with exercise had a synergistic effect on improving anti-stress capacity.

We found for the first time that trimetazidine induced the expression of HSP70 in mice, an effect which was similar to that of exercise, a protective effect on myocardium after EE. Some studies have shown that in an obesity and insulin resistance model, HSP70 induced by heat treatment, lipoic acid, and transgenic overexpression is similar to that of exercise 31. Our research showed exercise increased the expression of HSP70 significantly, which is consistent with studies showing that aerobic and resistance exercise induces could also upregulate the expression of HSP70 17, 18, 32, 33.

To gain further insight into whether the anti-stress capacity induced by exercise and trimetazidine is related to HSP70, we detected the expression of HSP70 12 hours' post-EE. Exercise and trimetazidine, alone or combined all increased the expression of HSP70 12 hours post-EE. Several studies have reported that HSP70 protected the heart immediately and 24 hours post-exercise, with HSP70 induced by short-term exercise also having a protective effect against myocardial I/R injury 34-36. In the current research, we found that exercise and trimetazidine alone or combined reduced the myocardium injuries caused by EE accompanied by the upregulation of HSP70.

Interestingly, we found that the expression of HSP70 was proportionate to the span of swimming time to exhaustion, suggesting that, the greater the HSP70 expression, the stronger the anti-stress capacity. In the present study, exercise and trimetazidine or their combination promoted the expression of HSP70, after which the myocardium of mice in these groups suffered limited damage compared with the control group after EE. This is similar to previous findings that HSP70 expression in fatal hyperthermal effects such as burns and fire accidents are correlated with survival time, as HSP70 expression is higher in the pulmonary and renal tissue of long-term survivors 37. Y Liu *et al*. also showed that during prolonged exercise training, as HSP70 increases, CK decrease in human skeletal muscle. The isolated hearts of transgenic mice overexpressing HSP70 protected mice from ischemia in different models 38, 39. As mentioned above, exercise and trimetazidine alone or in combination upregulate the expression of HSP70 which can be considered an indication of repairing and counteracting myocardial damage, indicating a potential protective effect of HSP70.

We observed that exercise and trimetazidine alone or combined improved autophagy and the expression of HSP70 simultaneously. One study reported that autophagy participates in exercise-induced cardioprotection via the HSP70/BAG3 complex 40. Trisha *et al*. showed that heat shock response after resistance exercise may partially regulate autophagy through HSP70 41. Studies indicated that drug preconditioning relieves cerebral infarction by up-regulating the expression of LC3-II and HSP70 in the ischemic area and HSP70 has a neuroprotective effect on ischemic injury by up-regulating the expression of autophagy in the cerebral ischemic area 20, 42, 43, suggesting that HSP70 expression is involved in autophagosomes formation and provided neuroprotection. So, we deducted that exercise combined with trimetazidine enhances the anti-stress capacity is related to the upregulation of autophagy and HSP70.

## Conclusions

This study explored the response of autophagy and HSP70 in the myocardium to exercise and trimetazidine during the recovery from EE for the first time. The current data indicates that exercise and trimetazidine, alone or combined, enhances the anti-stress capacity of mice. The underlying mechanism may be associated with the promotion of autography and the expression of HSP70.

## Figures and Tables

**Table 1:** Primer Sequence

HSP70	Forward	ACGTGGCCTTCACCGACACC
	Reverse	TGCACCACCGCATCGCCGAAC
GAPDH	Forward	GCGACTTCAACAGCAACTCCC
	Reverse	CACCCTGTTGCTGTAGCCGTA

## Abbreviations

EE: exhaustive exercise
BNIP3: BCL2/adenovirus E1B 19 kDa protein-interacting protein 3
LC3-II: microtubule-associated protein 1 light chain 3
P62: SQSTM1, sequestosome
HSP70: heat shock protein 70
HSPs: heat shock proteins
Mit: mitochondria
AV: autophagic vacuole
disc: myocardial disc
CK: creatine kinase
H/R: hypoxia/reoxygenation
